# Nanoparticle-Based Drug Delivery Systems for Anticancer Therapy: Microenvironment-Driven Strategies, Advances, and Challenges

**DOI:** 10.3390/pharmaceutics18101273

**Published:** 2026-10-08

**Authors:** Mengxin Ban, Sifang Zhao, Ziyu Liu, Lingling Zhao

**Affiliations:** 1 School of Materials Science and Chemical Engineering, Ningbo University, Ningbo 315211, China; banmengxin1015@163.com (M.B.); 18839966929@163.com (S.Z.); 2 Beijing Advanced Innovation Center for Biomedical Engineering, School of Engineering Medicine, Beihang University, Beijing 100191, China

**Keywords:** nanoparticles, drug delivery, anticancer therapy, targeting delivery, ATR principle

## Abstract

Cancer remains a major global health challenge, and nanoparticle-based drug delivery systems have emerged as a promising strategy for targeted cancer therapy. However, clinical translation is still limited by an incomplete understanding of how nanoparticles navigate the tumor microenvironment (TME) throughout the delivery process. This review presents an integrated, stage-by-stage framework for nanoparticle design that addresses the TME barriers encountered during systemic circulation, tumor-targeted enrichment, and drug release. During systemic circulation, PEGylation, hydrophilic polymer modification, cell membrane- or exosome-based biomimetic technologies, and biomolecular corona formation are employed to prolong circulation half-life. In the tumor-targeting stage, charge-reversal surfaces, ligand conjugation, ligand shielding, and tuning of physical properties enhance tumor accumulation and cellular internalization. For drug release, molecular and structural design enables intracellular responsive release, stepwise extracellular-to-intracellular release, and sequential release to distinct target cells. Beyond summarizing these strategies, we highlight the newly proposed active transport and retention (ATR) principle as a unifying mechanistic framework that explains how nanoparticles achieve tumor accumulation through active endothelial transport and retention via tumor component interactions. We further discuss translational barriers including complex fabrication, scalability hurdles, and biocompatibility constraints, and outline emerging solutions such as novel nanoparticle fabrication methods (microfluidics, turbulent jet, and microfluidic mixing), engineered biomimetic exosomes, organ-on-a-chip evaluation platforms, and AI-driven design screening. By connecting stage-specific design strategies to the ATR framework, this review offers a mechanistic foundation for the rational design of next-generation nanomedicines.

## 1. Introduction

Tumors are a major public health issue worldwide, having a significant impact on human life. According to GLOBOCAN 2022 estimates from the International Agency for Research on Cancer (IARC), a WHO agency, there were approximately 19.98 million new cases of malignant tumors worldwide in 2022, an increase of nearly 700,000 compared with 2020 [1,2]. The number of deaths from malignant tumors was 9.74 million, making it one of the leading causes of death worldwide. At present, the main treatment methods for malignant tumors include chemotherapy, radiotherapy, and surgical treatment [3,4]. In recent years, new treatment methods such as photothermal therapy, photodynamic therapy, immunotherapy, and gene therapy have gradually emerged [5].

**Figure 1:**
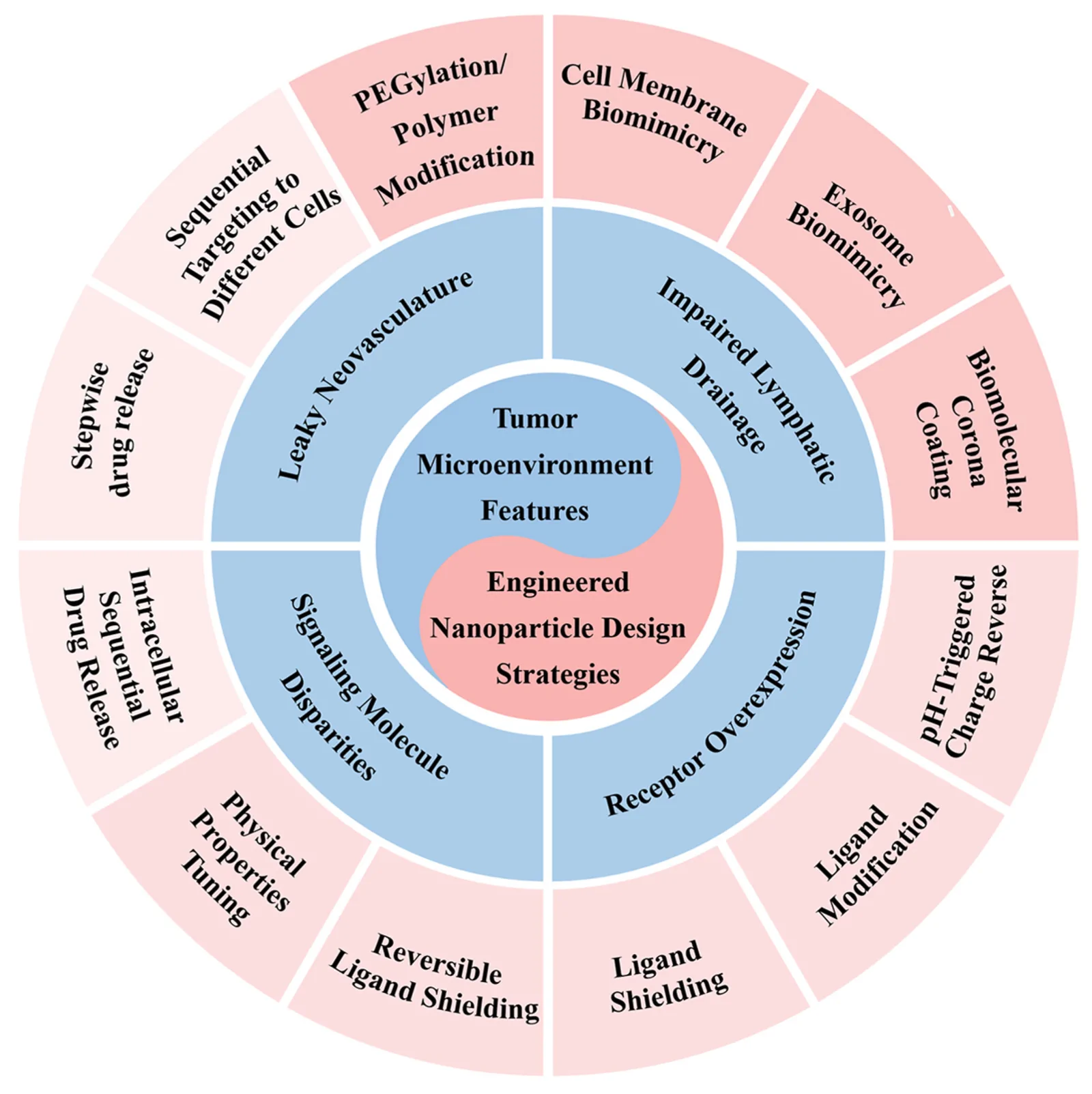
Tumor microenvironment features and corresponding engineered nanoparticle design strategies for anticancer drug delivery. The inner circle summarizes key TME features including leaky neovasculature, impaired lymphatic drainage, receptor overexpression, and signaling molecule disparities (e.g., acidic pH, elevated ROS, high GSH, hypoxia). The outer pink circle presents nanoparticle design strategies grouped by delivery stage and distinguished by color shades: prolonging circulation (dark pink), enhancing tumor accumulation and cellular internalization (medium pink), and targeted drug release (light pink).

The ultimate goal of tumor treatment is to deliver a sufficient amount of drugs into the tumor cells while minimizing the damage to normal tissues. However, whether it is conventional chemotherapy drugs such as cisplatin and oxaliplatin, or new photosensitizers and immunosuppressants, they do not have tumor specificity [6]. After being injected systemically, the accumulation rate of these drugs at the tumor site is low. To ensure efficacy, the dosage is often increased, inevitably causing systemic toxicity. Therefore, tumor-targeted drug delivery can greatly improve the therapeutic effect and reduce toxic side effects. Nanocarriers can increase drug loading, and can deliver drugs to the tumor site via the enhanced permeability and retention (EPR) effect due to the leakage structure of tumor new blood vessels [7], thus providing a new dimension for tumor-targeted treatment and being considered the most promising tumor treatment strategy [8]. Although the prospects are promising, the two inherent limitations of nanomedicine carriers severely affect their efficacy in treating metastatic tumors. Firstly, due to the recognition by the reticuloendothelial system (RES), the circulation time of nanomedicines is restricted, reducing their concentration in tumor tissues and making it insufficient to kill cancer cells [9]. Secondly, due to the obstruction of various biological and pathological barriers, the tumor penetration ability of nanoparticles is poor, which significantly limits their therapeutic effect [10]. For many years, researchers have been seeking new technologies and methods for nanoparticle design and modification to increase their tumor accumulation rate and thereby enhance their anti-tumor therapeutic effect. In this review, we first outline, in light of tumor microenvironment characteristics, current strategies and methods for the design and modification of nanoparticles at different stages of drug delivery, including blood circulation, tumor accumulation, cellular uptake, and drug release (Section 2 and Section 3; Figure 1). We then discuss recent mechanistic advances in nanoparticle entry into tumors, transitioning from the EPR effect to ATR principle, and elaborate on ATR-guided nanoparticle design strategies, including active transport direction control and intracellular retention, active transport triggering via surface engineering, active transport triggering via size tuning, and immune cell-mediated active transport and retention (Section 4). Finally, we discuss the translational barriers of nanoparticles—complex fabrication, scalability hurdles, and biocompatibility constraints—and outline emerging solutions such as novel production methods, engineered biomimetic exosomes, organ-on-a-chip evaluation platforms, and AI-driven design screening (Section 5).

## 2. Features of the Tumor Microenvironment

**Figure 2:**
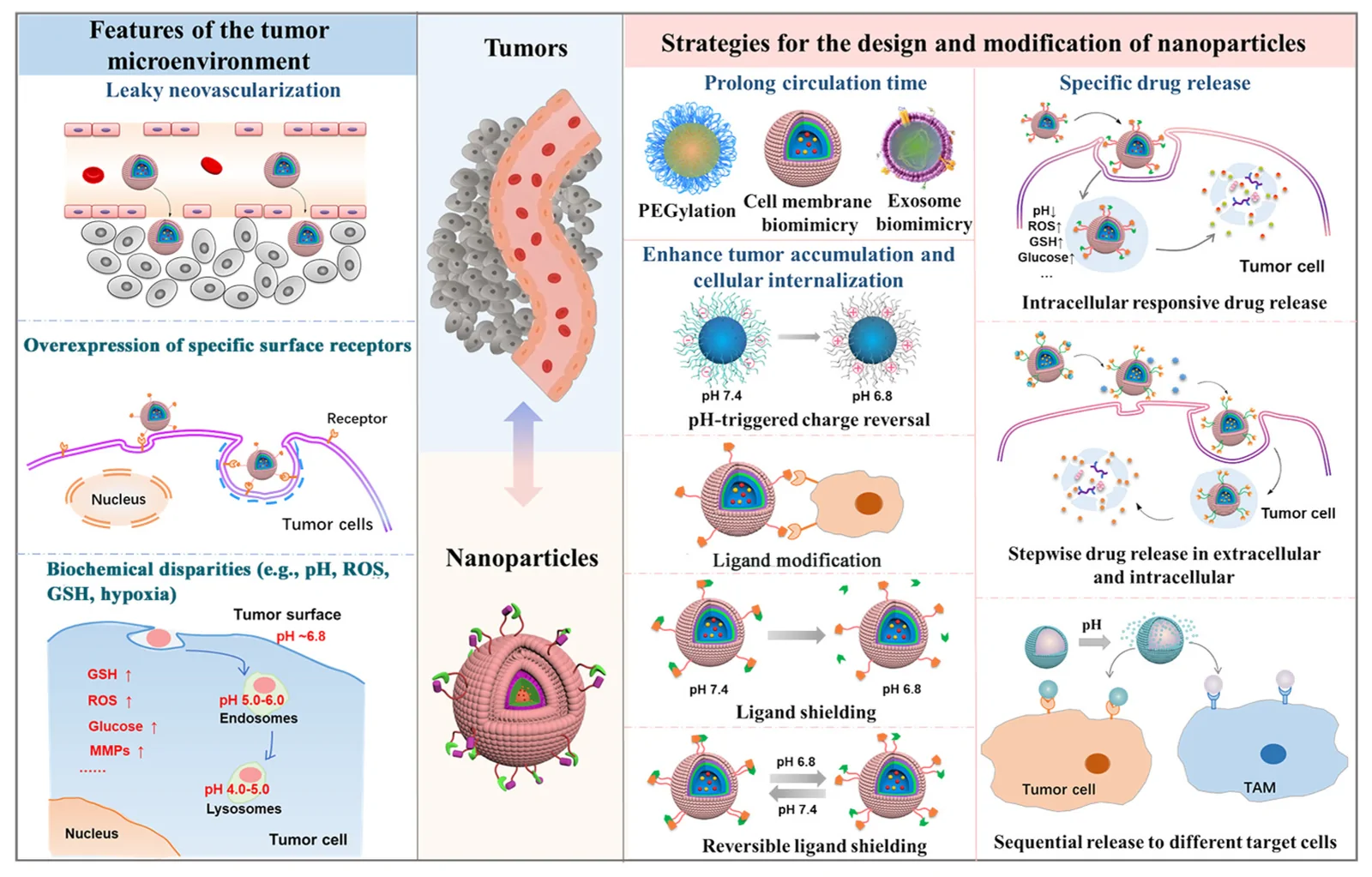
Tumor microenvironment features and their implications for nanoparticle design strategies.

During tumor progression, the interplay between cancer stem cells and their surroundings establishes a TME that is distinct from normal tissue [11]. In solid tumors, this is characterized by irregular neovascularization, featuring discontinuous blood vessels with wide endothelial gaps and absent lymphatic drainage [12]. The TME also exhibits significant biochemical alterations, such as acidic pH, elevated reactive oxygen species (ROS), high glutathione (GSH) levels, hypoxia, and abnormal ATP concentrations [13]. Furthermore, specific receptors are often overexpressed and more active on tumor cells than on normal cells [14]. Collectively, these TME–normal differences underscore the potential of TME-responsive nanocarriers to improve tumor-specific drug accumulation, minimize systemic toxicity, and enhance therapeutic outcomes (Figure 2).

### 2.1. Leaky Neovascularization in Tumors

In normal tissues, the dense and structurally intact intercellular spaces of microvessels restrict the passage of large molecules and particles through the vessel walls [15]. In contrast, solid tumor tissues exhibit enhanced neovascularization with wider, more permeable, and less organized intercellular gaps in the vessel walls, coupled with impaired or absent lymphatic drainage. These structural and functional differences facilitate the preferential accumulation of particles ranging from approximately 50 to 150 nm in diameter within tumors, enabling targeted delivery [16]. This phenomenon, known as the EPR effect, serves as a fundamental principle for tumor-targeted drug delivery and a cornerstone of modern anticancer nanomedicine [17]. Following systemic administration, nanomedicines are designed to exploit the EPR effect to achieve higher intratumoral accumulation, thereby improving the efficacy and therapeutic index of conventional chemotherapeutic agents while reducing systemic toxicity.

### 2.2. Overexpression of Specific Receptors on Tumor Cell Surface

Compared to healthy cells, cancer cells display distinct chemical and morphological surface characteristics resulting from altered membrane structures [18]. Malignant tumor cells often overexpress specific surface glycoproteins and receptors. For example, folate receptor α is overexpressed in approximately 40% of cancers [19], while sialic acid (SA) is upregulated on many tumor cell surfaces and correlates with malignancy and metastasis [20]. Transferrin receptor 1 (TfR1) is highly expressed at both the blood–brain barrier and in brain tumor tissues, with its expression level closely associated with glioma pathological grade [21]. These tumor-specific surface features can be harnessed to develop diagnostic and therapeutic tools. By conjugating high-affinity ligands to nanoparticle surfaces, targeted delivery can be achieved through ligand-receptor recognition and subsequent receptor-mediated endocytosis, thereby enhancing cellular uptake and therapeutic efficacy of nanomedicines [22]. For instance, Hui et al. developed a phenylboronic acid (PBA)-functionalized covalent organic framework as a fluorescence sensor for accurate SA detection, enabling early diagnosis of ovarian cancer [23]. Similarly, Fan et al. designed nanocarriers based on human ferritin heavy chain (HFn) for glioma treatment [24]. By leveraging the differential TfR1 overexpression on blood–brain barrier endothelial cells and glioma cells, along with their distinct binding interactions with HFn, these nanocarriers successfully crossed the blood–brain barrier via TfR1-mediated endocytosis and subsequently targeted glioma cells, thereby significantly enhancing antitumor therapeutic outcomes.

### 2.3. Signaling Molecule Disparities in Tumors

The TME exhibits significant differences from the physiological conditions of normal tissues [11]. Composed of diverse cellular and non-cellular components, including endothelial cells, pericytes, immune cells, cancer-associated fibroblasts, cancer stem cells, extracellular matrix, and vasculature, the TME displays distinct biochemical features relative to normal cellular environments [13]. These include acidic pH, excessive ROS generation, high GSH concentration, and hypoxic condition, etc. [25]. While the TME is also heterogeneous across different tumors and tumor regions—posing challenges for universal nanocarrier design—the consistent differences between the TME and normal tissues offer a foundation for designing TME-responsive nanocarriers for drug delivery. Based on these differences, researchers have developed various TME-responsive nanocarriers, that enhance therapeutic efficacy, promote tumor-specific drug accumulation, and reduce systemic toxicity, demonstrating considerable potential in cancer treatment [13]. Zeng et al. constructed an amphiphilic GSH-responsive polymer by linking chitosan and octadecylamine through disulfide bonds, with the LinTT1 peptide and a nitric oxide (NO) donor conjugated to chitosan [26]. This polymer self-assembles into nanomicelles that encapsulate the chemotherapeutic agent doxorubicin for breast cancer therapy. The system exhibits GSH gradient-responsive drug release: in the extracellular tumor microenvironment (1 mM GSH), NO is released to alleviate hypoxia, while in the cytoplasm (10 mM GSH), both NO and doxorubicin are rapidly released to exert cytotoxic effects. This GSH-responsive nanomicelle system has shown enhanced antitumor efficacy in breast cancer models.

## 3. Strategies for the Design and Modification of Nanoparticles

The primary objective of cancer therapy is to deliver sufficient drugs to the tumor cells while minimizing the damage to normal tissues, which is also central to addressing off-target toxicity. To achieve precise drug delivery to tumor cells and ensure effective treatment, it is essential to precisely design the molecular composition and structure of nanoparticles and thus overcome the numerous physiological barriers encountered during the drug delivery process at different stages, including the blood circulation, tumor aggregation, and drug release.

### 3.1. Prolong the Circulation Time of Nanoparticles in the Bloodstream

For nanoparticles to reach the tumor site, entry into the bloodstream is the first essential step. Extending their circulation time and preventing rapid clearance are critical for their subsequent accumulation within tumors. In recent years, several strategies have been developed to prolong the circulation of nanocarriers, such as PEGylation and biomimetic coatings derived from cell membranes or exosomes (Table 1).

#### 3.1.1. PEGylation Strategy

A common strategy to prolong the circulation time of nanoparticles is surface modification with hydrophilic polymers, such as polysaccharides, polyethylene glycol (PEG), and its analogues [44]. PEG can effectively prevent the nanoparticle aggregation and protein adsorption through its surface shielding effect, thereby reducing rapid blood clearance and promoting preferential accumulation in tumors via the EPR effect [27]. However, PEGylation also suppresses electrostatic interactions with cells, reduces nanoparticle uptake, and significantly diminishes biological activity [28]. Moreover, repeated administration of PEGylated nanoparticles in the same animal has been reported to lead to loss of their long-circulating properties over time [29]. This undesirable phenomenon, known as the “accelerated blood clearance (ABC)” effect, may limit the clinical applicability of such systems.

#### 3.1.2. Alternative Hydrophilic Polymer Modification

Given the limitations of PEGylation discussed above—particularly the ABC effect and the hindrance of cellular uptake—researchers have explored alternative hydrophilic polymers that can provide comparable stealth properties while avoiding anti-PEG antibody recognition. Several classes of polymers have emerged as promising candidates, including polysarcosine (PSar), poly(2-oxazoline)s (POx), polyglycerol (PG), and zwitterionic polymers.

PSar, a polypeptoid with excellent biocompatibility and enzymatic degradability, has demonstrated prolonged circulation times and enhanced tumor accumulation compared to PEGylated nanoparticles [30]. In a comparative study, PSar-modified nanoparticles exhibited a blood circulation half-life of 36.9 h, notably longer than that of their PEGylated counterparts [30]. Importantly, PSar-based nanocarriers reduced complement activation, protein adsorption, and macrophage uptake, resulting in enhanced stealth behavior without inducing the ABC phenomenon [45]. Recent studies have further demonstrated that PSar decoration can facilitate cell endocytosis, overcoming a key limitation of PEGylation that hinders cellular uptake [46].

POx represents another extensively validated PEG alternative. Head-to-head comparisons have shown that substituting PEG-lipids with poly(2-ethyl-2-oxazoline) lipids in lipid nanoparticles improves repeat-dosing performance, with reduced clearance from the bloodstream and lower levels of anti-stealth lipid immunoglobulins compared to PEG-lipid formulations [31]. PG has also been shown to prevent the induction of the ABC phenomenon upon repeated administration, whereas PEGylated liposomes exhibited accelerated clearance under identical conditions [35]. In a separate study, linear PG-functionalized lipid nanoparticles exhibited negligible binding to IgG anti-PEG antibodies while achieving comparable mRNA transfection efficiency to PEGylated formulations [47].

Zwitterionic polymers, including poly(carboxybetaine) (PCB), poly(sulfobetaine), and poly(2-methacryloyloxyethyl phosphorylcholine), carry paired positive and negative charges within each monomer, forming a net-neutral, superhydrophilic shell that resists protein adsorption and confers low immunogenicity [36]. A key advantage over PEG is their ability to circumvent the ABC phenomenon. PCB- and poly(N-methyl-N-vinylacetamide)-modified liposomes avoided ABC by shunning immune reactions [48], and zwitterionic PEG@PEG-NH_2_ nanoparticles showed negligible liver enrichment even in the presence of anti-PEG antibodies, attributed to minimized complement and immunoglobulin adsorption [49]. PCB-conjugated LNPs (e.g., ALC-0315-PCB LNPs) also did not exhibit ABC, improving repeated-dose performance. Zwitterionic polymers thus represent a promising PEG alternative that combines stealth properties with reduced immunogenic liabilities [50].

More broadly, alternative hydrophilic polymers, including PSar, POx, PG, and zwitterionic polymers, can provide stealth properties comparable to or exceeding PEG while circumventing the immunogenicity and ABC issues associated with PEGylation. The selection of an appropriate stealth polymer should therefore consider not only circulation half-life but also immunogenicity, repeat-dosing performance, and compatibility with the intended therapeutic cargo.

#### 3.1.3. Cell Membrane Biomimicry Strategy

Recent studies have shown that cell membranes can be extracted and reorganized onto the surface of nanocarriers. Biomimetic nanoparticles camouflaged with various cell membranes (such as red blood cell membranes, macrophage membranes, and cancer cell membranes) have been widely used in nanomedicine for drug delivery [51]. Owing to the complex biological composition of the membrane coatings, these nanocarriers exhibit distinctive immunological properties [52]. Erythrocyte have attracted considerable interest as drug carriers due to their excellent biocompatibility, biodegradability, and low immunogenicity [53]. They contain abundant “self-markers,” including proteins, glycosides, and SA residues, which help to suppress immune clearance [32]. Xu et al. used erythrocyte membranes to camouflage the nanoparticles, which improved physiological stability, extended systemic circulation, and enhanced anticancer efficacy [54]. Macrophage membranes inherit the surface protein profile and biological interface characteristics of the source cells. Therefore, macrophage-membrane-camouflage can protect nanoparticles from immune phagocytosis while enabling targeted recognition of antigens, facilitating accumulation at inflammatory or tumor sites [33]. Cancer cell membranes express cell adhesion molecules with homotypic binding domains that mediate multicellular aggregation [34]. This homotypic affinity may confer tumor-homing ability and targeting specificity to cancer-membrane-coated nanoparticles. Furthermore, beyond single-layer coatings, engineered and hybrid membrane systems are being explored to achieve synergistic functionalities [55]. Therefore, cell-membrane-based biomimetic nanotechnology represents a promising strategy for improving the therapeutic performance of nanoparticle systems.

#### 3.1.4. Exosome Biomimicry Strategy

Exosomes are endogenous lipid vesicles, typically 30–150 nm in size, that are secreted by various cell types and carry diverse biological cargo including nucleic acids, proteins, lipids, and metabolites [56]. Their surface expresses molecules such as CD47, CD55, and CD59, which help them evade immune clearance and prolong circulation in the bloodstream [37]. By fusing with cell membranes, exosomes can transfer their contents and signals to recipient cells, modulating cellular functions and offering considerable potential as nanomedicine delivery vehicles [57]. Additionally, exosomes can be reconstituted and coated onto synthetic nanoparticles to create biomimetic nanoplatforms [58]. Hill et al. coated Prussian blue nanoparticles with exosomes derived from U-87 tumor cells, generating exosome-mimetic nanoparticles that showed enhanced targeting and therapeutic efficacy against glioblastoma compared to PEG-coated counterparts [59]. While early research focused mainly on mammalian exosomes, their clinical translation is hindered by challenges such as immune rejection, toxicity risks, and scalable production [60]. In recent years, plant-derived exosome-like nanoparticles (PELNs), which range from approximately 30 to 300 nm in size, have gained increasing attention in nanomedicine [38,61]. PELNs offer advantages including wider source availability, easier large-scale production, and lower extraction costs, making them promising for biomedical applications [39].

#### 3.1.5. Biomolecular Corona Strategy

Upon systemic administration, nanoparticles are immediately exposed to blood components, leading to the spontaneous formation of a biomolecular corona on their surface. This corona, composed of proteins, lipids, and other biomolecules, fundamentally alters the biological identity of nanoparticles and governs their fate in vivo [40]. The composition of the corona is determined by both nanoparticle physicochemical properties (size, charge, shape, and surface chemistry) and environmental factors (exposure time, temperature, and flow conditions) [41]. Understanding and controlling corona formation is therefore essential for the rational design of nanomedicines. The glycan-coated nanoparticles reported by Clemente et al. exemplify how surface chemistry can modulate corona composition while preserving receptor-binding functionality [62]. Importantly, the corona influences multiple stages of the delivery process. During circulation, opsonin adsorption (e.g., complement proteins and immunoglobulins) promotes recognition by the mononuclear phagocyte system, accelerating clearance and reducing tumor accumulation [43]. For cellular uptake, corona components can determine the internalization pathway—for example, albumin-coated nanoparticles preferentially utilize gp60-mediated transcytosis, while apolipoprotein-enriched coronas enhance receptor-mediated endocytosis [63]. For targeting, the corona can either interfere with ligand-receptor recognition or, conversely, confer endogenous targeting capability [64]. Recent studies have shown that specific plasma proteins (e.g., apolipoproteins) can direct nanoparticles to particular organs, offering a strategy for organ-selective delivery without synthetic ligands [42].

### 3.2. Enhance the Tumor Accumulation and Cellular Internalization

Nanoparticles in systemic circulation can reach tumors primarily through the EPR effect. However, tumor accumulation and retention are distinct from cellular internalization [65]. While nanoparticles can accumulate in the tumor interstitium and be retained through interactions with tumor components, efficient cellular uptake is a separate and essential step for intracellular drug delivery, as nanoparticles remaining in the extracellular matrix risk re-entering circulation [66,67]. To address this, several strategies have been developed to promote the intracellular accumulation of nanoparticles within tumors, such as pH-responsive charge-conversion, ligand modification, ligand-shielding, physical property-mediated cellular internalization and intratumoral transport barrier-circumventing (Table 2).

#### 3.2.1. pH-Triggered Charge Reversal Strategy

The surface charge of nanocarriers plays a critical role in enhancing tumor accumulation and cellular uptake [86]. Positively charged nanoparticles can bind to negatively charged tumor cell membranes, promoting internalization; however, they also tend to exhibit toxicity toward erythrocytes and are rapidly cleared from circulation [87]. Conversely, negatively charged nanoparticles can resist protein adsorption via electrostatic repulsion, prolong blood circulation [88]. To resolve this dilemma, researchers have developed nanocarriers with switchable surface charges. Taking advantage of the slightly acidic extracellular tumor microenvironment (pH 6.5–6.8) compared with normal tissues (pH 7.4), surface charge conversion can be triggered by pH-sensitive chemical groups introduced onto the nanocarrier surface. This allows the nanoparticles to remain negatively charged in the bloodstream and switch to a positive charge in the acidic tumor region, improving both stability and targeted delivery [68,69]. Various functional groups and bonds, such as acid-labile amides, benzoyl isocyanide bonds, amphoteric moieties and amino groups, have been employed in designing such pH-responsive charge-reversal systems [89]. Zou et al. developed a series of charge-reversible nanoparticles by reacting dimethylmaleic anhydride with polyglycerol-grafted graphene oxide carrying different densities of amino groups [70]. Their study revealed that the physiological stability and tumor cell uptake of nanoparticles depend on the amino density of the precursor. Nanoparticles derived from a high-density precursor were unstable at physiological pH, underwent rapid charge conversion, induced nonspecific interactions, and were cleared quickly, resulting in negligible tumor accumulation. In contrast, nanoparticles from lower-density precursors exhibited improved stability, delayed charge switching, extended circulation, and enhanced tumor accumulation.

#### 3.2.2. Ligand Modification Strategy

Ligand-modified nanomedicine delivery systems represent an effective strategy to enhance tumor accumulation and reduce systemic toxicity [90]. Tumor cells often overexpress specific surface biomarkers, including sialic acid (SA) residues, folate receptors, and SA receptors. By functionalizing nanoparticles with corresponding targeting ligands, receptor-mediated endocytosis can be leveraged to improve tumor-specific delivery [71,72]. Ge et al. prepared three types of nanoparticles with similar physicochemical properties but different surface ligands: phenylboronic acid-modified nanoparticles (PBA-NPs), phenylboronic acid/folate dual-modified nanoparticles (FA-PBA-NPs), and phenylboronic acid/mannose dual-modified nanoparticles (MAN-PBA-NPs) [73]. The results showed that the mannose-modified nanoparticles were preferentially internalized by macrophages, limiting their tumor accumulation. In contrast, PBA-NPs and FA-PBA-NPs achieved higher tumor accumulation (approximately 11.3% of the administered dose). Moreover, FA-PBA-NPs were taken up by about 30% of cancer cells, substantially higher than the 10–18% observed for PBA-NPs or MAN-PBA-NPs, leading to more pronounced antitumor efficacy.

#### 3.2.3. Ligand Shielding Strategy

While ligand-modified nanoparticles have shown promise in tumor-targeted therapy, the attached targeting moieties often trigger unintended immune recognition and clearance by the RES, which compromises their long-term circulation in the bloodstream [74]. For instance, although hyaluronic acid can bind to the CD44 receptor that is overexpressed on the surface of most tumor cells, other hyaluronic acid receptors (e.g., HARE and LYVE-1) are also abundant in the RES, leading to off-target uptake [75]. Similarly, while tumor cells overexpress certain surface molecules such as SA residues, normal tissues (particularly in the liver and lungs) also express SA groups to some extent, resulting in undesired accumulation [76].

Notably, the TME differs from normal tissues in several respects, including elevated protease levels, high interstitial pressure, leaky vasculature, hypoxia, and acidic extracellular pH. Exploiting these distinctions to design tumor-responsive ligand exposure systems may improve targeting specificity. Consequently, a growing research focus has been the development of nanoparticles whose targeting ligands remain shielded during circulation and are selectively exposed within tumors. Such a stimuli-responsive shielding/deshielding strategy can enhance nanoparticle stability in blood while promoting tumor-specific cellular uptake [77]. Typically, ligands are concealed in circulation by stealth coatings or cage-like groups and are revealed at the tumor site through cleavage of stimuli-sensitive linkers. For example, Long et al. developed a pH-dependent shielding system in which PBA ligands on nanoparticles were temporarily masked by coordinated fructose (Fru) under physiological conditions [78]. In circulation, the stable Fru-PBA complex prevented non-specific interactions. Upon reaching the acidic tumor microenvironment, the binding weakened, exposing PBA to bind SA residues overexpressed on tumor cells and thereby increasing tumor accumulation.

#### 3.2.4. Reversible Ligand Shielding Strategy

While the ligand-shielding strategy has improved tumor-specific targeting to some extent, studies indicate that only about 0.7% of the injected nanoparticle dose typically accumulates in solid tumors, a level that remains suboptimal [67]. In practice, not all nanoparticles that undergo deshielding at the tumor site are effectively internalized by cancer cells; those that fail to be taken up can re-enter systemic circulation [79]. However, once the shielding groups are removed in the tumor microenvironment, it is generally difficult to re-shield the exposed ligands upon re-entry into the bloodstream. This irreversible nature of most shielding approaches leaves deshielded nanoparticles vulnerable to recognition and clearance by the RES, ultimately limiting the overall effectiveness of the strategy. Cao et al. recently proposed a reversible ligand-shielding strategy by co-modifying the nanocarrier surface with two targeting molecules: phenylboronic acid (PBA) and galactose (Gal) [80]. Under physiological conditions, PBA and Gal form a mutual complex that effectively masks their targeting functions. In the acidic tumor microenvironment, this interaction weakens, allowing PBA to bind SA residues while Gal engages desialylated protein receptors on tumor cells. Compared with irreversibly shielded carriers, the reversible system exhibits a 48% longer blood half-life, 54% lower hepatic uptake, 40% higher tumor accumulation, and a 10.3% increase in tumor inhibition. Therefore, reversible ligand shielding offers a promising approach to balance circulation stability and tumor-specific targeting.

#### 3.2.5. Physical Property-Mediated Cellular Internalization Strategy

Beyond surface charge and targeting ligands, the intrinsic physicochemical properties of nanoparticles—composition, size, shape, and rigidity—profoundly influence cellular internalization and downstream biological effects [81,82]. Particle size can influence the endocytic pathway and uptake efficiency, although the preferred route depends on the cell type and particle system. For example, Rejman et al. observed that in non-phagocytic B16 cells, 50 nm fluorescent latex beads were internalized via clathrin-coated pits, whereas caveolae-mediated uptake became increasingly involved at larger sizes and predominated at 500 nm; clathrin-mediated uptake was limited to particles up to approximately 200 nm [91]. Whether similar size-dependent preferences apply to other cell types or particle systems remains to be established. Shape also plays a decisive role: rod-shaped nanoparticles generally exhibit higher uptake efficiency than spherical counterparts in certain cell types, although the optimal geometry is cell-type dependent [81,92]. Mechanical rigidity is another critical parameter: rigid particles tend to be internalized more efficiently via clathrin-mediated endocytosis, whereas soft particles may preferentially enter through macropinocytosis [93,94]. Composition further modulates uptake behavior, as the chemical nature of the stabilizing layer can govern internalization at sub-100 nm sizes [95]. These findings underscore the importance of considering the full spectrum of nanoparticle physical properties in the rational design of delivery systems.

#### 3.2.6. Intratumoral Transport Barrier-Circumventing Strategy

After extravasation, nanoparticles must traverse the tumor interstitium to reach cancer cells distant from blood vessels. However, intratumoral transport is severely impeded by the dense extracellular matrix (ECM) and elevated interstitial fluid pressure (IFP) [96]. The tumor ECM is enriched in collagen, hyaluronic acid, and fibronectin, forming a crosslinked network that restricts the convective and diffusive movement of nanoparticles [83]. Moreover, leaky vasculature and impaired lymphatic drainage elevate IFP, further reducing convective transport and forcing nanoparticles to rely primarily on slow diffusion [84]. Cabral et al. found that in highly permeable tumors, micelles of all tested sizes (30–100 nm) penetrated the tumor tissue, whereas in poorly permeable pancreatic tumor models, among the tested sizes, only 30 nm micelles achieved sufficient penetration for an antitumor effect, with larger micelles (50–100 nm) tending to remain perivascular [97]. These findings indicate that the size dependence of tumor penetration is context-dependent. In addition to particle size, tumor penetration can also be influenced by tumor type, vascular permeability, ECM density, IFP, as well as particle surface properties and flexibility. To overcome these barriers, strategies such as ECM degradation (e.g., hyaluronidase or collagenase), size-shrinking or charge-conversion systems, and modulation of IFP have been explored [85]. For example, enzyme-responsive nanoparticles that degrade hyaluronic acid in the tumor matrix have been shown to significantly improve deep tumor penetration and therapeutic efficacy [98]. These approaches complement the accumulation and internalization strategies discussed above and are essential for achieving uniform drug distribution throughout solid tumors.

### 3.3. Strategies for Specific Drug Release

To achieve therapeutic efficacy, nanoparticles need to release their cargo upon reaching the tumor site. However, the stability required during systemic circulation can impede drug release. Hence, promoting drug release within tumor cells while maintaining circulatory stability is crucial for enhancing therapeutic efficacy. Researchers have developed various strategies to achieve controlled drug release intracellularly and/or extracellularly, including stimuli-responsive intracellular release, stepwise extracellular-intracellular release, and sequential release to different target cells (Table 3).

#### 3.3.1. Endosome Escape and Intracellular Drug Release

For many antitumor agents, including chemotherapeutics and STING agonists, intracellular release is essential to achieve therapeutic efficacy [104]. While extracellularly released drugs may diffuse into tumor cells, a significant fraction is effluxed via P-glycoprotein-mediated multidrug resistance, leading to low bioavailability and reduced antitumor activity [105]. Moreover, even with enhanced cellular uptake facilitated by ligand-modified nanoparticles, insufficient drug concentrations within tumor cells often persist due to barriers such as incomplete drug release, membrane-associated multidrug resistance mechanisms, and inefficient transport to relevant subcellular compartments [106].

Endocytosis is a major pathway for nanoparticle cellular entry, initially trapping nanoparticles within endosomal vesicles [107]. Since the therapeutic activity of these agents typically occurs in the cytosol or other non-lysosomal compartments, efficient endosomal escape is critical for the success of intracellular-targeted therapies [108]. This step is widely regarded as a major bottleneck in nanoparticle-based delivery systems [108], particularly for lipid nanoparticle-mediated nucleic acid delivery [109]. Consequently, researchers are actively exploring new approaches to fundamentally elucidate the mechanisms of endosomal escape. Current strategies commonly involve the use of materials responsive to the intracellular microenvironment, such as ionizable lipids [110], pH-sensitive membrane-disruptive polymers or peptides [111], as well as lysosomotropic agents (e.g., chloroquine) and endosomal trafficking inhibitors (e.g., bafilomycin A1) [112]. Another approach involves photochemical degraders, such as photosensitizers, which generate singlet oxygen radicals to disrupt lysosomal membranes [113].

pH-responsive release is an effective strategy for promoting drug release within cells due to the differences in pH value in the endosomes/lysosomes, cytoplasm and blood [114]. The pH of blood is approximately 7.4, whereas the cytoplasmic pH is slightly lower (around 7.2) [115]. In contrast, the pH values within endosomes and lysosomes are much lower, ranging from 5.5 to 6.8 and 4.0 to 5.0, respectively [116]. The pKa of the imidazole group in histidine is approximately 6.0, thus histidine can act as a “proton sponge” to absorb protons in the acidic environment of the endosome, causing the endosomal membrane to expand and rupture. Therefore, the introduction of histidine fragment into the polypeptide sequence can effectively promote the endosome escape [117]. Recent study has reported a new type of charge-reversal biomimetic nanosystem for targeted therapy of tumors [99]. The surface charge of the polymer nanoparticles coated by erythrocyte membrane changed from negative to positive under the trigger of the low pH value in the tumor cell endosomes/lysosomes. The oppositely charged polymer cores and the erythrocyte membranes cause the collapse of the lipid bilayer, further lead to the destruction of the red blood cell membrane and promote the intracellular drug release, thereby enhancing the therapeutic effect of anti-glioma treatment.

Additionally, disulfide bonds within nanoparticles may undergo covalent binding with extracellular sulfhydryl groups, potentially inducing membrane deformation that enhances both cellular uptake and endosomal escape [118]. Recent studies further indicate that PBA modification significantly promotes nanoparticle lysosomal escape and facilitates trans-cellular transport [119]. This effect is hypothesized to arise from specific interactions between PBA groups and lysosomal membrane proteins or heat shock proteins. Ye et al. developed a PBA-modified poly-L-lysine dendrimer (PBA-DGL) for siRNA delivery [100]. Under acidic endosomal conditions, PBA modification strengthens polymer-membrane interactions, leading to membrane disruption and enhanced endosomal escape.

#### 3.3.2. Sequential Drug Release

In tumor combination therapy, due to the differences in physical and chemical properties, pharmacokinetics, and tissues distribution of the drugs, as well as the time sensitivity of therapeutic targets, sequential or programmed administration is often required [120,121]. The release of different drugs in tumor cells and their synergistic effects is a common method to improve the therapeutic effect of tumors. Yang et al. developed a glutathione-sensitive and paclitaxel-loaded zinc-iron oxide biomimetic nanosystem for the activation and enhancement of the STING signaling pathway through the synergistic effect of Fe^3+^, Zn^2+^ and paclitaxel within cells [101]. Zinc ferrite nanoparticles were camouflaged with cancer cell membranes, enabling the nanosystem to target tumor cells. Due to the high concentration of glutathione within the tumor cells, the zinc ferrite nanoparticles would decompose to release Fe^3+^ and Zn^2+^. The Fe^3+^ would induce the production of ROS within the cells and lead to DNA leakage, while the Zn^2+^ would promote the separation of cGAS-DNA molecules and enhance the activity of the cGAS enzyme. Additionally, the continuous release of paclitaxel would inhibit the mitosis of tumor cells, further strengthening the activation of the cGAS/STING pathway. The nanosystem promoted the maturation of dendritic cells, increased the infiltration of immune cells, and reshaped the immunosuppressive tumor microenvironment, thus achieving satisfactory immunotherapy effects with minimal systemic toxicity in the tested animal models.

#### 3.3.3. Stepwise Drug Release

Stepwise release in extracellular and intracellular to regulate tumor microenvironment barriers can significantly enhance the therapeutic effect of tumor treatment. Zhang et al. developed a size-tunable, dual-responsive system based on ATP-modified platinum nanoparticles for programmed drug release and enhanced antitumor immunity [102]. An MMP-2-sensitive nanocluster was constructed by co-encapsulating the IDO1 inhibitor NLG919 with ATP-conjugated platinum nanoparticles (ABP). In the MMP-2-rich tumor microenvironment, the nanocluster disintegrates, releasing small-sized ABP for deep tumor penetration. Under acidic conditions, ATP is released, exposing PBA sites that bind to tumor cell surface sialic acid for targeted recognition. ATP also serves as an “eat me” signal to dendritic cells, initiating an antitumor immune cascade. Intracellular ROS triggers Pt(II) release from BP, which catalyzes H_2_O_2_ decomposition into O_2_ and •OH, alleviating hypoxia and generating positive feedback to further promote Pt(II) release. This process amplifies the cancer immunity cycle and enhances CTL infiltration. NLG919 reverses the immunosuppressive tumor microenvironment, synergistically inhibiting primary tumor growth, distant tumors, and pulmonary metastasis.

#### 3.3.4. Sequential Release to Distinct Target Cells

The TME comprises tumor cells and regulatory components such as tumor-associated macrophages (TAMs), which constitute up to 50% of solid tumor mass and critically influence tumor progression [122]. Thus, sequentially targeting different TME components for drug release and synergistic effects is essential for enhancing antitumor efficacy. Sun et al. constructed a pH-sensitive reversible-shielding nanoplatform that enables targeted delivery of BLZ-945 to TAMs and platinum nanoparticles to tumor cells, achieving synergistic chemo-immunotherapy [103]. The system consists of two components: dextran-modified PLGA nanoparticles (DNPs) loaded with BLZ-945, and ultrasmall phenylboronic acid-modified platinum nanoparticles (PBA-Pt) covalently linked to dextran via pH-responsive borate ester bonds. Under acidic tumor conditions, PBA-Pt dissociates from DNPs. DNPs then target TAMs and release BLZ-945 release to alleviating the immunosuppressive within TME. Meanwhile, PBA-Pt penetrates deeply into tumor tissue, binds to SA residues on tumor cells, and releases intracellular Pt(II), inducing immunogenic cell death. Collectively, these actions promote antigen release, dendritic cell maturation, cytotoxic T lymphocyte infiltration, and a potent antitumor immune response.

## 4. ATR-Guided Nanoparticle Design Strategies

### 4.1. Mechanisms of Nanoparticle Delivery to Solid Tumors: From EPR to ATR

Efficient delivery of nanoparticles into solid Tumors is a key prerequisite for precision anticancer therapy. Understanding the biological mechanisms that govern nanoparticle transport to solid Tumors can guide the rational design of nanocarriers, thereby improving delivery efficiency and facilitating clinical translation. The current delivery mechanism, known as the EPR effect, was established in 1986 when the research groups of Jain and Maeda independently demonstrated the preferential accumulation of proteins and dyes in VX2 carcinoma and sarcoma 180 ascites tumor models [7,123]. According to this principle, nanoparticles passively extravasate into tumors through interendothelial gaps and are retained due to impaired lymphatic drainage [124]. However, nanoparticles designed based on the EPR effect have demonstrated limited efficacy in delivering therapeutic agents to solid tumors (0.7% injected dose), and high clinical failure rates [67,125].

Recently, Warren C. W. Chan and coworkers proposed an alternative mechanism, termed the ATR (active transport and retention) principle [65]. This principle posits that nanoparticles enter tumors via active endothelial transport processes (e.g., transcytosis, vesiculo-vacuolar organelle-mediated transport, and immune cell migration) [126], are retained within the tumor through interactions with tumor components, and subsequently exit through lymphatic vessels [65]. Using four different mouse models, three distinct types of human tumors, mathematical simulations and modeling, and two complementary imaging techniques, they demonstrated that interendothelial gaps are not the primary route for nanoparticle entry into solid tumors. Instead, up to 97% of nanoparticles enter tumors through active endothelial processes [127]. They identified a subset of tumor endothelial cells, termed nanoparticle-transporting endothelial cells (N-TECs), that facilitate this process [128]. Their findings revealed that only 21% of tumor endothelial cells, located on a minority of blood vessels, are involved in transporting nanoparticles into the tumor microenvironment. N-TECs act as gatekeepers, determining the entry point, distribution, cellular accessibility, and quantity of nanoparticles that extravasate into the tumor. Compared to other tumor endothelial cells, N-TECs exhibit higher expression levels of genes associated with nanoparticle transport and vascular permeability. Once inside the tumor, nanoparticles exit via intratumoral or peritumoral lymphatic vessels, with the dominant exit mechanism depending on nanoparticle size. Nanoparticles that exit the tumor through the lymphatic system are returned to the bloodstream, allowing them to recirculate and potentially interact with the tumor again in subsequent passes [65]. The introduction of this new principle is poised to reshape engineering strategies for particle and material design in tumor delivery, providing a novel framework for the development of nanomedicines for cancer treatment and diagnosis.

In contrast to the EPR effect, which provides clear design rules for optimizing nanoparticles, namely a size smaller than endothelial gaps and prolonged circulation [129,130], the ATR principle is not universally applicable. It suggests that no single ideal nanoparticle exists; rather, the optimal design is dictated by the transport process to the target disease site. Nanoparticles with an ideal size for entry may exhibit poor retention, while those optimized for prolonged circulation may show suboptimal cellular interactions. Moreover, these behaviors can vary significantly across different tumor types [66]. Consequently, advancing cancer nanomedicine and its clinical translation, as well as elucidating how nanoparticles should be engineered for medical use, requires a deeper mechanistic understanding of nanoparticle delivery to solid tumors. This necessitates integrating multiple delivery mechanisms and leveraging machine learning tools to unravel the complex nano–bio interactions that occur throughout the delivery process.

### 4.2. Active Transport Direction Control and Intracellular Retention

Such designs intervene at two ATR stages: inhibiting apical exocytosis and promoting basal transport to direct transport, and terminating transcytosis to achieve intracellular retention. One of the core rationales underlying the ATR principle proposed by Chan and coworkers is the recognition that, after nanoparticles cross the tumor vascular endothelium, apical exocytosis can shuttle them back into the bloodstream, while excessive and indiscriminate transcytosis leads to insufficient intratumoral retention [131]. To address this issue, Yang K et al. developed the PG@BAM-LRC biomimetic micro–nano system [132]. Platelet membrane camouflage (PG) prolongs circulation time and confers tumor vascular homing capacity, followed by MMP-9 triggers release of liposomes (LRC) and berbamine (BAM). BAM inhibits apical exocytosis of nanoparticles via Rab8 regulation, promoting basal transport; R8 enhances endothelial uptake, and Golgi-targeting Cys ligand directs endosome–Golgi transport to bypass lysosomal degradation. In tumor cells, furin cleaves RKKR-Cys, terminating transcytosis and retaining nanoparticles intracellularly. Transwell assays showed nearly all LRC crossed bEnd.3 monolayers and were retained in downstream 4T1 cells. In orthotopic PANC-1 tumors with weak EPR, this system achieved optimal accumulation and efficacy. This “promote forward transport, inhibit reverse exocytosis, terminate excessive penetration” three-stage ATR regulation offers a notably comprehensive ATR engineering framework.

### 4.3. Active Transport Triggering via Surface Engineering

The core logic of surface-engineering-triggered active transport is to actively trigger or enhance endothelial transcytosis via dynamic regulation of surface chemistry (e.g., charge reversal, ligand modification, zwitterionic switching), with the trigger signal originating from the nanoparticle surface [133]. Cao et al. constructed cascade-responsive zwitterionic polyprodrug micelles P(OC7A-DOX), in which tumor microenvironment (hypoxia/weak acidity) triggers charge reversal of the POC7A shell, switching the surface from stealth to positively charged [134]. This system integrates charge reversal-triggered active transport with cascade-responsive release, enabling deep penetration/retention and intracellular drug release, thereby overcoming CAPIR (Circulation, Accumulation, Penetration, Internalization, Release) barriers for efficient tumor therapy. Zheng et al. reported CRGDK peptide-modified P1 micelles that employ a more refined dual-pathway sequential transcytosis strategy [135]. First, CRGDK binding to NRP-1 mediates receptor-mediated transcytosis to achieve initial penetration; subsequently, charge reversal is triggered in the acidic microenvironment, activating a second cation-mediated transcytosis pathway that synergistically promotes deep tumor infiltration. This carrier also integrates mitochondria-targeting function, releasing DOX and cinnamaldehyde within mitochondria under ROS response, achieving precise delivery at the subcellular organelle level.

Notably, mechanistic studies underlying the ATR principle also rely on precise regulation of surface engineering parameters. Chan and coworkers, using transmission electron microscopy and in vivo microscopy, found that nanoparticles cross tumor endothelium mainly via non-receptor-mediated macropinocytosis, which contributes 76.9–95.1% of transendothelial transport and involves macropinocytic vesicles of 200 nm or larger; membrane ruffles on tumor endothelial cells capture circulating nanoparticles, which are internalized via macropinocytosis into intracellular vesicles and subsequently released into the tumor interstitium [136]. This mechanism provides a direct constraint for surface-engineering-triggered ATR design: surface engineering should preferentially enhance nanoparticle interaction with membrane ruffles and promote macropinocytic capture, rather than relying solely on receptor-mediated endocytosis or passive leakage through endothelial gaps.

### 4.4. Active Transport Triggering via Size Tuning

ATR-guided design typically focuses on the selection of surface ligands (e.g., targeting transferrin receptor, gp60, etc.) [137] or the regulation of surface charge [138], whereas size is often regarded as an auxiliary parameter to be optimized. Wang et al. constructed a series of dendritic dots, Gx-Ac (x = 5–8) with a ~2 nm size gradient (4.9–11.1 nm) and constant surface chemistry, isolating size as the sole variable [139]. They found that when the size increased from G7-Ac (ca. 8.7 nm) to G8-Ac (ca. 11.1 nm), transcytosis-mediated tumor entry efficiency increased 7-fold. In transcytosis blockade experiments, the fluorescence signal of G8-Ac decreased by 81.0%, whereas that of G7-Ac decreased by only 12.3%, clearly revealing the existence of a size threshold. Further mechanistic studies showed that this efficiency jump originated from a size-dependent switch in endocytic pathways: smaller particles mainly relied on passive diffusion and inefficient endocytosis, whereas 11.1 nm particles triggered an efficient endothelial transcytosis pathway. Functionally, G8-Ac achieved deep subcutaneous tumor penetration and crossed the blood-brain tumor barrier to target orthotopic gliomas, outperforming smaller analogues. In the Gx-Ac series, the surface chemistry was constant (all acetylated cyanine cores), and the only variable was geometric size. This means that the active transport triggering mechanism revealed in this study is not achieved through surface chemical engineering, but indirectly through size—a physical parameter—that selectively regulates cellular uptake pathways. It reveals another route to achieve active transport within the ATR framework: size tuning as an independent design dimension beyond surface engineering.

### 4.5. Immune Cell-Mediated Active Transport and Retention

Singh et al. systematically compared EPR and ATR paradigms and identified active endothelial transport, including immune cell-mediated transport, as the defining feature of ATR [140]. In immune cell hitchhiking, both transport and retention are mediated by immune cells rather than nanoparticle properties. The main challenge is that internalized nanoparticles may remain trapped in carrier cells. These designs exploit immune cell chemotaxis for active transport and tumor residence for retention. Gao et al. reported a representative bacteria-mimetic system [141]. Gold nanoparticles (GNPs) modified with β-cyclodextrin (CD-GNPs) and adamantane (ADA-GNPs) were coated with Escherichia coli outer membrane vesicles (OMVs) to form M-GNPs. After intravenous injection, OMVs confer a bacteria-like surface, enabling selective phagocytosis by circulating immune cells. Intracellularly, OMVs degrade in lysosomes, exposing CD and ADA groups that drive GNP self-assembly into ~2.5 μm aggregates via host–guest interactions. This aggregation yields dual benefits: reduced exocytosis (~32.5% efflux) for enhanced retention, and near-infrared absorption via plasmon coupling for photothermal conversion. Immune cells then carry the aggregates to melanoma via inflammatory chemotaxis. A positive feedback loop was designed: initial photothermal therapy enhances local inflammation, recruiting more immune cell carriers for a second treatment. This work achieves in situ immune cell hitchhiking without ex vivo engineering, eliminating autologous cell isolation and individualization limits. The intracellular self-assembly also provides a new retention strategy: locking nanoparticles within carrier cells to reduce leakage, rather than relying solely on passive interactions with tumor components.

## 5. Challenges and Future Perspectives

In summary, precise drug delivery and effective tumor treatment require nanomedicines to successfully navigate multiple interconnected biological stages. The integration of these sequential functions into a single nanocarrier is important for achieving significant therapeutic outcomes. While nanoplatforms hold substantial promise in antitumor therapy, their clinical translation continues to face considerable challenges (Table 4).

### 5.1. Challenges and Opportunities in Nanoplatforms Preparation

To overcome physiological delivery barriers and achieve precise tumor targeting, nanoplatforms require meticulous design at both molecular and structural levels [153]. This frequently leads to complex, multilayered architectures and labor-intensive, multi-step preparation processes, hindering large-scale industrial production. Conventional methods for preparing lipid nanoparticles, such as bulk mixing, detergent dialysis, and conventional rapid mixing (e.g., manual or T-junction mixing), as well as polymer nanoparticle techniques, including solvent evaporation, salting out, dialysis, and microemulsion, generally involve multiple steps, offer limited control over particle properties, and result in inconsistent sizes [154]. Therefore, simplifying synthesis procedures and advancing novel fabrication technologies are critical for facilitating the translation and clinical application of nanomedicines.

Microfluidics offers a powerful approach for preparing lipid nanomedicines [155]. This technology manipulates minute fluid volumes within microfabricated channels, providing advantages in miniaturization, integration, and automation [156]. Compared to traditional fluidic systems, microfluidic platforms enable precise control over transport and reaction kinetics at reduced length and time scales through carefully engineered channel geometries [157]. This allows for superior control over nanoparticle properties, including size, distribution, morphology, and surface functionality [158]. Moreover, microfluidics supports scalable production from small-batch screening to GMP-compliant manufacturing, revolutionizing the synthesis of lipid nanoparticles and advancing lipid-based nanomedicine development. Jiang et al. utilized microfluidics to fabricate hierarchically structured nanoparticles by precisely regulating fluid velocity in microchannels, creating a chemiluminescence immunosensor for highly sensitive detection of carcinoembryonic antigen [142]. Maeki et al. developed a one-step microfluidic method to produce exosome-like lipid nanoparticles (ELNPs) with tunable composition and surface properties [143]. By functionalizing ELNPs with exosomal proteins, they demonstrated that ITG αVβ5 modification significantly improved RNA delivery efficiency both in vitro and in vivo. This approach elucidates exosomal protein functions and provides a versatile tool for advancing targeted delivery systems and exosome research.

While microfluidics offers precise control over nanoparticle properties, the choice of fabrication method itself critically influences the physicochemical and biological characteristics of the resulting nanoparticles. A systematic comparison of turbulent jet mixing, microfluidic mixing, and extrusion for producing biomimetic nanoparticles demonstrated that turbulent jet and microfluidic mixing yield superior stability, higher membrane protein incorporation, improved batch-to-batch reproducibility, and enhanced targeting, whereas extrusion leads to diminished performance due to shear-induced protein loss [159]. Similarly, for mRNA lipid nanoparticles, different mixing techniques—including turbulent-flow, microfluidic, and manual pipetting—produce particles with widely different physical properties and biological performance, with manual pipetting generating particles that do not resemble those produced by turbulent-flow mixers used at commercial scale [160]. This discrepancy between bench-scale and commercial-scale mixing techniques means that laboratory results may not be directly replicable using materials manufactured at scale, underscoring the importance of selecting fabrication methods that are both reproducible and scalable from the outset.

Among the various nanoplatforms, lipid nanoparticles (LNPs) have gained prominence, particularly for mRNA delivery [161]. A typical LNP consists of four components: an ionizable lipid, a helper lipid (e.g., DSPC), cholesterol, and a PEG-lipid [162,163]. The ionizable lipid is critical for nucleic acid encapsulation and endosomal escape, while the PEG-lipid controls particle size and circulation time [162]. LNPs are commonly formulated by rapid mixing or microfluidic mixing, which enables reproducible particle size and encapsulation efficiency [164]. Despite their success, LNPs face translational challenges including batch-to-batch variability, long-term storage stability, immunogenicity of the PEG-lipid, and the need for scalable manufacturing under GMP conditions [161,164]. These challenges are shared with other nanoplatforms and emphasize the need for continued innovation in formulation and manufacturing.

From a clinical translation perspective, several nanomedicines have already been approved, including Doxil (PEGylated liposomal doxorubicin), Abraxane (albumin-bound paclitaxel), Onivyde (liposomal irinotecan), and COVID-19 mRNA-LNP vaccines [125,161,164]. These successes demonstrate the feasibility of nanomedicine but also highlight the main barriers to broader clinical use, including complex multi-step fabrication, batch-to-batch variability, long-term storage instability, PEG-lipid immunogenicity, limited availability of certified biocompatible materials, and difficulties in GMP-scale manufacturing. Regulatory evaluation of multifunctional nanoplatforms remains challenging, and standardized protocols, such as those established by the Nanotechnology Characterization Laboratory (NCL) and the National Cancer Institute (NCI), are therefore essential.

### 5.2. Challenges and Opportunities in Nanoplatforms Biocompatibility

Biocompatibility is a critical determinant of the safety and efficacy of nanoplatforms for clinical translation [165]. The clinical transformation of nanocarriers is highly dependent on biocompatibility and requires optimization from multiple dimensions such as material design, immune regulation, and metabolic pathways [166]. Standardized evaluation systems, such as the protocols established by the NCL, which was founded by the NCI in collaboration with the FDA and NIST, are essential for advancing this process. Currently, to improve clinical feasibility, commonly selected raw materials include those derived entirely from biological sources or those with prior FDA approval for biocompatibility. However, the range of certified materials remains relatively limited and often falls short of meeting the design requirements of multifunctional nanosystems. Therefore, developing new biocompatible materials and establishing more efficient, standardized evaluation frameworks is of significant importance for accelerating the translation and application of nanosystems.

Green nanotechnology is revitalizing nanobiology, with exosomes emerging as promising therapeutic agents and delivery platforms. Through endogenous or exogenous loading strategies, engineered exosomes can be modified to improve targeting, evade immune clearance, and flexibly encapsulate cargo, demonstrating significant clinical potential [144]. However, scaling up production for clinical applications remains a key challenge. Recent advances in synthetic biology have introduced biomimetic exosomes as a viable alternative. These vesicles are produced via methods such as extrusion, artificial synthesis, or liposome fusion, and they can mimic the functions of natural exosomes in drug delivery research [145]. Compared to natural exosomes, biomimetic versions provide over 100-fold higher yields, enhanced biocompatibility, and reduced batch variability. Moreover, programmable biomimetic exosomes engineered through chemical or genetic approaches closely resemble natural exosomes in size, morphology, and surface protein markers, while allowing controlled, large-scale production [146].

Organoid and organ-on-a-chip technologies represent significant advances in biomedical research by offering physiologically relevant human models [167]. Organoids are three-dimensional miniature tissues cultured from stem cells or specific cell types in vitro, capable of simulating the structure, function, and genetic profiles of real organs and maintaining these properties over extended culture periods [168]. When integrated with microfluidic systems to form organoids-on-a-chip, these models allow precise control of the tissue microenvironment, vascularization, and inter-tissue communication, while enabling high-throughput analysis [147]. Such integrated platforms improve the accuracy of predicting human physiological and pathological responses, providing an effective system for toxicity assessment and drug screening of nanoplatforms. Tumor organoids closely recapitulate in vivo tumor architecture and drug sensitivity, accelerating drug testing, reducing costs, and enhancing translational efficiency. A prominent case is the use of a colorectal cancer organoid biobank to identify the bispecific antibody MCLA-158 for selective tumor cell killing [148]. MCLA-158 subsequently received FDA authorization to proceed with a Phase 1 clinical trial in April 2018 [169]. The European Union has recognized organoids as a valid alternative to certain animal experiments, and clinical trials such as “organoid-guided therapy” (NCT06268652) are underway to assess their clinical utility. Du et al. developed a vascularized patient-derived tumor organoid (PDTO) chip that reconstructs tumor-vascular interactions and supports long-term monitoring [149]. Their findings highlight the crucial role of the Notch pathway in tumor cell migration, with PDTO chip behavior strongly correlating with clinical metastatic potential, making this platform a valuable tool for metastasis research and preclinical drug evaluation.

The rapid development of artificial intelligence (AI) has brought significant innovation to biomedicine [170]. AI enables the prediction and simulation of the biological effects of nanomaterials [171]. Compared to conventional experimental approaches, AI-assisted prediction can rapidly screen promising nanomaterials and identify interaction patterns between nanomaterials and biological systems, thereby reducing unnecessary experiments and lowering research costs and time [150]. AI analysis provides deeper insights into the complex mechanisms underlying nanomaterial-biological interactions, establishing a theoretical foundation for optimizing nanomaterial design and enhancing biological system functionality. Saldinger et al. developed NeCLAS, a versatile and efficient machine learning pipeline that predicts nanoscale interaction sites and reproduces interactions in both biological and non-biological systems while delivering interpretable predictions [151]. Mi et al. used machine learning models and AI algorithms to predict nanoparticle delivery efficiency to tumors and major organs based on nanoparticle physicochemical properties, treatment strategies, and dosage parameters [152]. They found that a deep neural network model exhibited superior predictive performance and can serve as a screening tool for designing nanoparticles with high tumor-targeting efficiency. AI driven approach also contributes to reducing or partially replacing the use of experimental animals, thus accelerating the development and clinical translation of nanomedicines.

## 6. Conclusions

This review outlines current strategies for nanoparticle design and modification based on tumor microenvironment characteristics across the drug delivery process, including blood circulation, tumor-targeted enrichment, and drug release. At each stage, PEGylation/hydrophilic polymer modification, biomimetic membrane/exosome technologies, and biomolecular corona formation address circulation stability; charge reversal, ligand conjugation, ligand shielding, and tuning of physical properties enhance tumor accumulation and internalization; and molecular/structural design enables intracellular, stepwise, and sequential drug release. Furthermore, we discuss ATR-guided nanoparticle design strategies, including active transport direction control and intracellular retention, active transport triggering via surface engineering, active transport triggering via size tuning, and immune cell-mediated active transport and retention. Despite these advances, clinical translation remains hindered by complex fabrication, scalability challenges, and biocompatibility constraints. Therefore, simplifying synthesis, developing novel biomaterials, and establishing standardized evaluation are essential. Promising tools include novel nanoparticle fabrication methods, engineered and programmable biomimetic exosomes, organ-/organoid-on-a-chip models for predictive toxicology, and AI-driven design screening. Collectively, these advances position nanoparticle-based delivery systems as a promising platform for cancer therapy.

## Figures and Tables

**Table 1:** The advantages and disadvantages of the commonly used strategies for prolonging the blood circulation time of nanocarriers.

Strategies	Advantages	Disadvantages	Ref.
PEGylation	Prevent aggregation of the nanoparticle and protein adsorption due to the surface shielding effect.	1. Inhibited the electrostatic interaction between cells and the uptake of the nanomedicines. 2. Accelerated blood clearance in repeated injections.	[27,28,29]
Alternative Hydrophilic Polymer Modification	1. Avoid PEG immunogenicity: reduced anti-PEG antibodies, ABC phenomenon, and hypersensitivity. 2. Low immunogenicity/antifouling: zwitterionic polymers, PSar, and PG can reduce protein adsorption and complement activation.	1. PSar: Difficult excretion of high-molecular-weight; Repeated dosing may still induce ABC. 2. PG: Hyperbranched PG requires high surface density for PEG-equivalent stealth; Linear PG can still induce anti-PEG antibodies, risking immune cross-reactivity. 3. Zwitterionic polymers: Insufficient long-term stability; Anti-polyelectrolyte effect. 4. POx/PEOZ: Protein conjugation may reduce activity.	[30,31]
Cell Membrane Biomimicry	1. Erythrocyte membranes: Inhibit immune attacks due to the “self-markers”. 2. Macrophage membranes: Protect from being engulfed by immune cells, and can accurately recognize antigens to target inflammatory tissues or tumor sites. 3. Cancer cell membranes. Tumor homing ability and targeting capability.	Limited source, and ethical concerns in clinical use.	[32,33,34,35,36]
Exosome Biomimicry	1. Evade immune clearance. 2. Plant-derived exosomes have wide raw material sources, easy large-scale production, and low extraction costs.	Mammals-derived exosomes face clinical translation obstacles, such as potential immune rejection, toxicity risks, and production challenges.	[37,38,39]
Biomolecular Corona Strategy	1. Enables endogenous targeting and organ-selective delivery. 2. Allows modulation of cellular uptake pathways. 3. Surface chemistry can modulate corona composition while preserving receptor-binding functionality.	1. Composition is complex and difficult to control precisely. 2. Accelerates clearance and reduces tumor accumulation. 3. May interfere with active targeting. 4. Alters the biological identity of nanoparticles.	[40,41,42,43]

**Table 2:** The advantages and disadvantages of the commonly used strategies for enhancing the tumor accumulation and cellular internalization of nanoparticles.

Strategies	Advantages	Disadvantages	Ref.
pH-triggered charge reversal strategy	Nanomedicines have negatively charged surfaces in the bloodstream to inhibit protein adsorption, and convert to positively charged surfaces at the tumor site to facilitate internalization.	The toxicity of the detached small molecules needs to be considered.	[68,69,70]
Ligand modification strategy	Enhance tumor cell internalization through receptor-mediated endocytosis.	1. Lead to incorrect immune recognition and clearance by the RES. 2. Lead to off-target effects because some receptors are also expressed to a certain extent in normal tissues.	[71,72,73,74,75,76]
Ligand shielding strategy	The targeting ligands are shielded in the blood circulation and de-shielded in the tumor region, thus enhancing the tumor targeted accumulation.	When the non-internalized nanoparticles return to the bloodstream, it is difficult to re-shield the ligands in the blood, thus easily recognized and eliminated by the RES.	[77,78,79]
Reversible ligand shielding strategy	1. The dual ligands form complexes to shield their targeting ability from each other in the blood, and perform de-shielding to enhance the tumor-targeting ability of nanoparticles in tumor sites. 2. The mutual shielding-deshielding effect between the ligands is reversible.	There are relatively few types of ligands that possess this reversible shielding and deshielding function.	[80]
Physical Property-Mediated Cellular Internalization Strategy	1. Provides multiple independent tunable parameters beyond surface charge and targeting ligands. 2. Enables predictable control over endocytic pathways via particle size.	1. Optimal geometry is cell-type dependent. 2. Multi-parameter optimization increases design complexity.	[81,82]
Intratumoral Transport Barrier-Circumventing Strategy	1. Directly targets the key physical barriers of intratumoral transport. 2. Offers multiple actionable approaches. ECM degradation, size-shrinking or charge-conversion systems, and IFP modulation have been explored to overcome these barriers.	1. The tumor ECM forms a formidable crosslinked network. 2. Elevated IFP further limits convective transport. 3. Size-dependent trapping remains a major obstacle.	[83,84,85]

**Table 3:** Strategies for intracellular and/or extracellular drug release.

Drug Release Behavior	Strategies	Ref.
Intracellular drug release	1. pH-triggered lysosomal escape. 2. PBA-enhanced lysosomal escape.	[99,100]
Sequential drug release	The release of different drugs in tumor cells and their synergistic effects, e.g., glutathione-triggered release of Fe^3+^ to induce ROS production and DNA leakage, release of Zn^2+^ to activate the cGAS/STING pathway, and continuous release of paclitaxel to inhibit the mitosis of tumor cells.	[101]
Stepwise drug release	Stepwise drug release in extracellular and intracellular to enhance therapeutic effect, e.g., MMP-2-triggered release of NLG919 to reverse the tumor immunosuppressive microenvironment and small sized ABP to achieve deep penetration into the tumor, pH-triggered release of ATP as “eat me” signal in extracellular, and ROS-induced release of Pt(II) within the cells to induce immunogenic cell death (ICD).	[102]
Sequential release to different target cells	Sequential targeting to different cells for drug release and synergistic effects, e.g., nanoparticles dissociated into two parts in the acidic microenvironment of tumors, one part targets to TAMs and release BLZ-945 to alleviate the TIM, and the other part targets to tumor cells and release Pt(II) to induce ICD.	[103]

**Table 4:** Challenges and opportunities for nanoplatforms in clinical translation and application.

Aspects	Challenges	Opportunities	Ref.
Preparation method	Rather cumbersome and often requires multiple steps of synthesis due to the relatively complex structure.	Microfluidic technology can prepare multi-level structured nanoparticles with better performance control in a much simpler way.	[142,143]
Biocompatibility	1. The existing certified material types are relatively limited and cannot meet the design requirements of multi-functional nanoplatforms. 2. The standard certification process is cumbersome and time-consuming.	1. The development of new materials with good biocompatibility using green technology, such as engineered exosomes, biomimetic exosomes, and programmable exosomes. 2. The development of organoid and organ-on-a-chip (OOAC) technologies offer a more efficient and accurate platform for toxicity assessment and screening on the nanoplatforms. 3. AI prediction and simulation can quickly screen out potential nanomaterials and reduce unnecessary experiments and lower research costs and time.	[144,145,146]
			[147,148,149]
			[150,151,152]

## Data Availability

No new data were created or analyzed in this study. Data sharing is not applicable to this article.

## Abbreviations

The following abbreviations are used in this manuscript:

EPR	Enhancing permeability and retention
RES	Reticuloendothelial system
TME	Tumor microenvironment
ATR	Active transport and retention
IARC	International Agency for Research on Cancer
PSar	Polysarcosine
POx	Poly(2-oxazoline)s
PG	Polyglycerol
PCB	Poly(carboxybetaine)
ROS	Reactive oxygen species
GSH	Glutathione
ATP	Adenosine triphosphate
SA	Sialic acid
TfR1	Transferrin receptor 1
HFn	Human ferritin heavy chain
NO	Nitric oxide
PEG	Polyethylene glycol
PELNs	Plant-derived exosome-like nanoparticles
PBA	Phenylboronic acid
PBA-NPs	Phenylboronic acid-modified nanoparticles
FA-PBA-NPs	Phenylboronic acid/folate dual-modified nanoparticles
MAN-PBA-NPs	Phenylboronic acid/mannose dual-modified nanoparticles
Fru	Fructose
Gal	Galactose
ABP	ATP-conjugated platinum nanoparticles
DNPs	Dextran-modified PLGA nanoparticles
TAMs	Tumor-associated macrophages
N-TECs	Nanoparticle-transporting endothelial cells
ELNPs	Exosome-like lipid nanoparticles
ICD	Immunogenic cell death
PDTO	Patient-derived tumor organoid
AI	Artificial intelligence
